# Effect of Long Working Hours on Self-reported Hypertension among Middle-aged and Older Wage Workers

**DOI:** 10.1186/s40557-014-0025-0

**Published:** 2014-09-03

**Authors:** Dong Hyun Yoo, Mo-yeol Kang, Domyung Paek, Bokki Min, Sung-il Cho

**Affiliations:** 1 Department of Occupational and Environmental Medicine, Graduate School of Public Health and Institute of Health and Environment, Seoul National University, Gwanak-ro 1, Seoul 151-742, Gwanak-gu, Republic of Korea; 2 Department of Preventive Medicine, School of Medicine, Seoul National University, Daehakro 103, Seoul 110-799, Jongno-gu, Republic of Korea

**Keywords:** Long working hours, Hypertension, Self-reported hypertension, Wage workers

## Abstract

**Objectives:**

Many studies have reported an association between overwork and hypertension. However, research on the health effects of long working hours has yielded inconclusive results. The objective of this study was to identify an association between overtime work and hypertension in wage workers 45 years and over of age using prospective data.

**Methods:**

Wage workers in Korea aged 45 years and over were selected for inclusion in this study from among 10,254 subjects from the Korean Longitudinal Study of Ageing. Workers with baseline hypertension and those with other major diseases were excluded. In the end, a total of 1,079 subjects were included. A Cox proportional hazards model was used to calculate hazard ratios and adjust for baseline characteristics such as sex, age, education, income, occupation, form of employment, body mass index, alcohol habit, smoking habit, regular exercise, and number of working days per week. Additional models were used to calculate hazard ratios after gender stratification.

**Results:**

Among the 1,079 subjects, 85 workers were diagnosed with hypertension during 3974.2 person-months. The average number of working hours per week for all subjects was 47.68. The proportion of overtime workers was 61.0% (cutoff, 40 h per week). Compared with those working 40 h and less per week, the hazard ratio of subjects in the final model, which adjusted for all selected variables, working 41-50 h per week was 2.20 (95% confidence interval [CI], 1.19–4.06), that of subjects working 51-60 h per week was 2.40 (95% CI, 1.07–5.39), and that of subjects working 61 h and over per week was 2.87 (95% CI, 1.33–6.20). In gender stratification models, the hazard ratio of the females tended to be higher than that of the males.

**Conclusion:**

As the number of working hours per week increased, the hazard ratio for diagnosis of hypertension significantly increased. This result suggests a positive association between overtime work and the risk of hypertension.

## Introduction

Uehata coined the term “karoshi” (sudden death from overwork) in the 1980s [1],[2]. He analyzed cases of occupational accidents undergoing counseling, 70.1% of which involved fatigue-related deaths, and found that the primary cause was stroke or cardiac disease due to long working hours [3]. Concerns about long working hours and research on the associated risks subsequently increased in Japan [1]. The situation in the Republic of Korea (hereafter, “Korea”) is not different. In 2011, the average number of working hours per year was 2,090, which is second-highest among OECD countries, so long working hours is emerging as a social issue in Korean society [4].

Long working hours may have various adverse effects. For example, overwork is known to be associated with mortality [5], cardiovascular disease [2],[6], diabetes mellitus [7], hypertension [8]-[10], metabolic syndrome [11], work disability [12], and depression [13].

Among these adverse effects, hypertension is important with respect to public health because it is closely and directly related to vascular mortality [14], and is a major cause of stroke [15]. Worldwide, 7.6 million premature deaths (about 13.5% of the global total) and 92 million disability-adjusted life years (6.0% of the global total) were attributed to high blood pressure [16]. In particular, the prevalence of hypertension is 30% among individuals ≥30 years of age in Korea [17]. About 35% of cases of cerebrovascular disease and 21% of cases of ischemic heart disease were attributed to hypertension in Korea in one study [18].

Along with the increased awareness of these problems, epidemiological research suggests that long working hours may be an important amenable risk factor for hypertension [8],[19]. However, research results on the health effects of long working hours have been inconclusive. Yang et al. [20] reported a positive association between work hours and hypertension in the California working population. Hayashi et al. [9] showed that the 24-hour average blood pressure of the overtime groups was higher than that of the control groups. However, several studies showed a negative association. Nakanishi et al. [21] and Wada et al. [22] reported that the risk of hypertension decreased as working hours increased. Moreover, some studies have not found any association. Park et al. [23] identified a relationship between overtime work and certain cardiovascular function parameters in a field survey of 238 male engineers in Korea. However, the authors did not observe the effect of long working hours on blood pressure. Sorensen et al. [24] and Pimenta et al. [25] also could not show any association between long working hours and the risk of hypertension.

Due to inconclusive evidence on this topic, difficulties have arisen in terms of improving working conditions and worker compensation policies. Against this background, we explored the relationship between the number of working hours and self-reported hypertension using prospective data.

## Materials and methods

### Subjects

This study used a sample from the first- to third-wave data of the Korean Longitudinal Study of Ageing (KLoSA) conducted by the Korea Labor Institute and Korea Employment Institute Information Service. Research was conducted in 2006, 2008, and 2010. In 2006, 15 major cities and provinces were selected by stratification, and 1,000 households were randomly selected. Of these 1,000 selected households, successful interviews were conducted in 6,171 households, and the total number of surveyed subjects was 10,254. These subjects underwent biennial follow-up.

The interviewers visited each house with a laptop computer. They instructed the subjects to read questions and then input the answers by themselves. The first wave of interviews was conducted from August through December 2006, the second wave from July through November 2008 and the third wave from October through December 2010.

**Figure 1:**
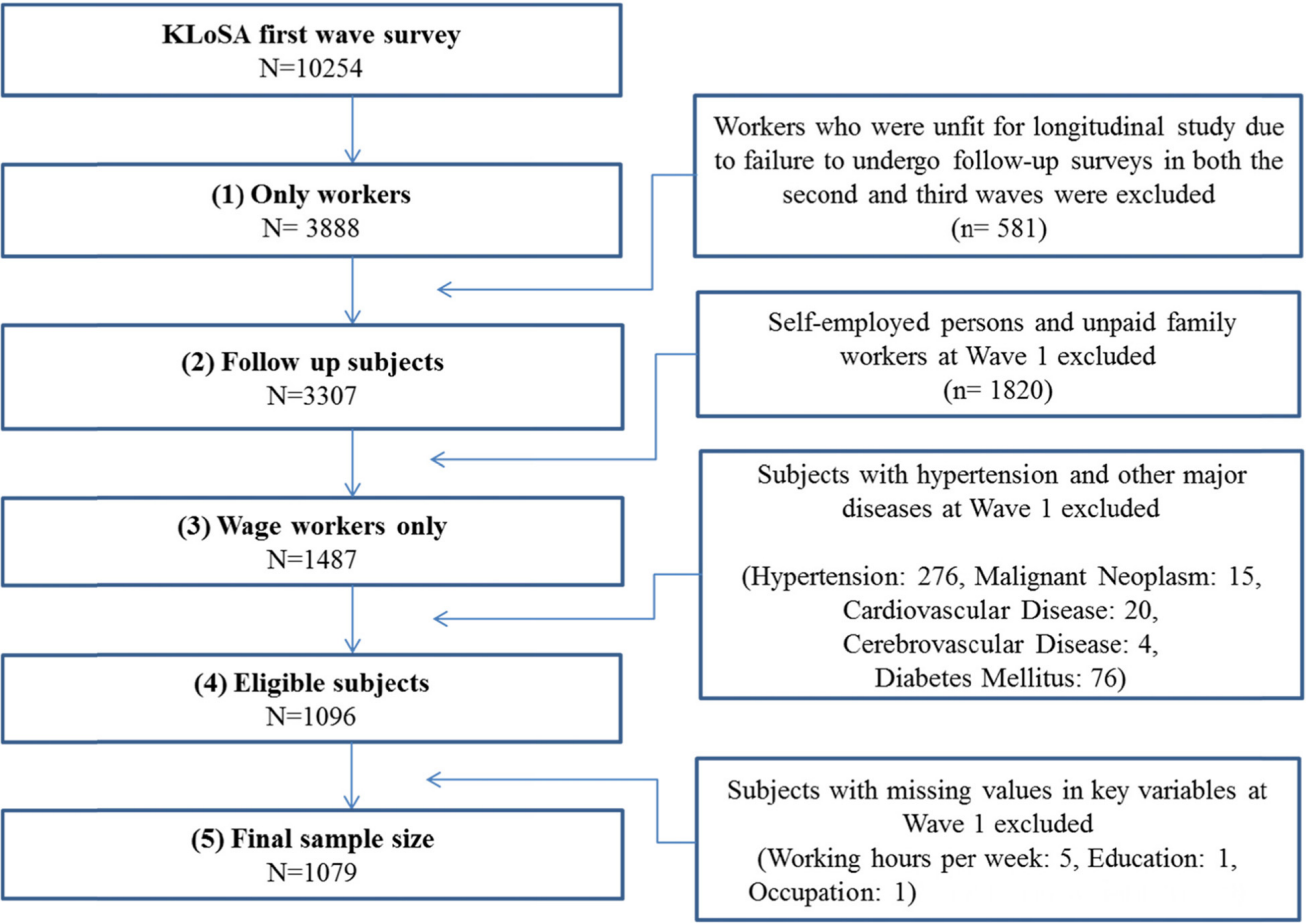
Schematic diagram of the study population.

A total of 3,888 workers were selected from among the 10,254 subjects selected in the first wave, and 581 who were unfit for longitudinal study due to failure to undergo follow-up surveys in both the second and third waves were excluded. We also excluded 1,820 self-employed workers or unpaid family workers because this study only considered wage workers. In addition, subjects with hypertension (n = 276) or major diseases such as malignant neoplasia (n = 15), cardiovascular disease (n = 20), cerebrovascular disease (n = 4), or diabetes mellitus (n = 76) were excluded. Moreover, subjects with missing data with respect to the number of hours worked per week (n = 5), education level (n = 1), occupation (n = 1), and height and weight (n = 10) were eliminated. In the end, 1,079 subjects were included in the study (Figure 1).

### Study variables and measurements

#### Self-reported hypertension and onset time

Self-reported hypertension and the date of diagnosis, both of which were important variable for this study, were measured by the questionnaire responses. We excluded subjects who answered “Yes” to the question “Has a doctor ever told you that you have high blood pressure or hypertension?” in the first-wave questionnaire by regarding them as having a medical history of hypertension. New cases of hypertension were defined as those individuals who reported physician-diagnosed hypertension in one of the follow-up questionnaires and who did not have hypertension at baseline. The question was as follows: “Have you been diagnosed with hypertension by a doctor since the last survey?” The subsequent question, “In what year and month was your hypertension first diagnosed?” determined the diagnosis date of hypertension during the follow-up period.

#### Follow-up period

In subjects diagnosed with hypertension, the follow-up period was calculated as the difference between the date of the first survey and the date of hypertension diagnosis. In normotensive subjects, the follow-up period was calculated from the date of the first survey to that of the third survey. If normotensive subjects were lost to follow-up in the second or third wave, their follow-up period was calculated as the difference between the date of the first survey and that of the final survey that they completed.

#### Working hours and overtime work

We obtained data from the first wave in 2006. The information on self-reported working hours was collected in the first-wave questionnaire by the following question: “How many hours do you work per week, excluding mealtimes?” Working hours per week were categorized as ≤40, 41–50, 51–60, and ≥61 hours. We defined ‘overtime work’ as over 40 hours per week with reference to the 40 h outlined in the Labor Standards Act in Korea.

#### Other covariates

The KLoSA questionnaire includes questions about a wide array of characteristics. Using the questionnaires, we measured several covariates that were considered necessary for this study. Data were collected on demographic characteristics (age, sex, and education), regular exercise, smoking habit, alcohol habit, and body mass index (BMI; height and weight), all of which are reported risk factors of hypertension. We also collected data on the number of working hours per week, occupation type, form of employment (regular, temporary, or part-time), and income, all of which are occupational-related factors.

The education level was categorized as elementary school graduation or less, middle school graduation, high school graduation, and college graduation or more. An exercise habit was defined as exercise more than once a week and categorized as regularly performed or otherwise. A smoking habit was categorized as current smoking, past smoking, and nonsmoking. A drinking habit was categorized as current drinking and nondrinking. BMI was calculated by height and weight, which subjects filled in by themselves, and was calculated as weight/height^2^ (kg/m^2^); a BMI of ≥25.0 kg/m^2^ was regarded as overweight. Jobs were categorized into eight groups by job type. Jobs were categorized by form of employment into regular, temporary, or part-time. Income was categorized as ≤990,000 won, 1,000,000–1,990,000 won, 2,000,000–2,990,000 won, ≥3,000,000 won, and neglect to answer. Finally, the number of working days per week was categorized as ≤5 and ≥6.

#### Statistical analyses

The major focus of the data analysis was the relationship between the onset of self-reported hypertension and the number of working hours per week. We calculated the mean number of working hours and the proportion of overtime workers for each categorized variable to analyze the general characteristics of the subjects. We then analyzed the distribution of each group by Pearson’s chi-squared test or the Kruskal–Wallis test. Cox proportional hazards models were used to evaluate the association between the number of working hours per week and the onset of self-reported hypertension, and weighted cases were used. Assumptions regarding the use of Cox proportional hazards were met by inspection of the log minus log function at the covariate mean [26]. The models comprised Model 1 (not adjusted), Model 2 (adjusted for age and sex), Model 3 (adjusted for age, sex, and health-related factors including smoking habit, alcohol habit, regular exercise, and BMI), and Model 4 (adjusted for age, sex, health-related factors, education, and occupational factors including income, occupation, form of employment, and working days per week). Additional models, Model 1_s_- Model 4_s_, were calculated after gender stratification. Model 1_s_ was not adjusted. Model 2_s_ was adjusted for age. Model 3_s_ was adjusted for age, and health-related factors. Model 4_s_ was adjusted for age, health-related factors, education, and occupational factors. The analysis was performed using IBM SPSS Statistics, version 20.0.0 software (IBM, Inc., Armonk, NY, USA) A two-sided probability value of p < 0.05 was considered to indicate statistical significance.

## Results

Table 1 shows the general characteristics of the selected population. The mean number of working hours per week among all subjects was 47.68 ± 14.21 h per week. The proportion of overtime workers was 61.0% (cutoff, 40 h per week). Male subjects comprised 65.2%, and workers aged 45 to 49 years comprised the largest proportion, and beyond that, with increasing age, the groups comprised decreasing proportions of the total. Workers with a high school diploma comprised the highest percentage, at 39.9%, and those with a college degree or beyond comprised the lowest, at 19.7%. The group that had finished middle school was associated with the highest average number of working hours at 50.41 ± 15.96 h, and those with a college diploma or beyond were associated with the least, at 43.86 ± 10.08 h. With respect to income, 1,000,000–1,990,000 won was associated with the highest and ≤990,000 won with the second highest number of working hours. Among occupations, the engineers and semi-experts were associated with the lowest and service and sales workers with the highest number of working hours. Workers with regular employment, as opposed to those with temporary or part-time jobs, were the largest group, comprising 69.3% of the total, and the working hours of those with regular employment averaged 48.56 h, which was the highest among the workers. With respect to BMI, only 19.4% of all subjects were in the high-risk group of >25 kg/m^2^.

**Table 1:** General characteristics of the selected population

**Characteristics**		**n (%)**	**Number of working hours per week**	**Overtime workers n (%)**
**Sex**				
	Male	703(65.2)	47.37 ± 13.53	417(59.3)
	Female	376(34.8)	48.26 ± 15.41	241(64.1)
**Age (years)**				
	45–49	441(40.9)	47.23 ± 12.71	271(61.5)
	50–54	311(28.8)	47.15 ± 13.46	176(56.6)
	55–59	174(16.1)	47.76 ± 14.07	109(62.6)
	60–64	93(8.6)	48.94 ± 18.68	61(65.6)
	≥65	60(5.6)	51.53 ± 19.73	41(68.3)
**Education**				
	Elementary school graduation or less	218(20.2)	48.05 ± 17.40	135(61.9)
	Middle school graduation	217(20.1)	50.41 ± 15.96	165(70.0)
	High school graduation	431(39.9)	48.01 ± 12.82	275(63.8)
	College graduation or beyond	213(19.7)	43.86 ± 10.08	96(45.1)
**Income (won)**				
	≤990,000	312(28.9)	45.67 ± 17.65	178(57.1)
	1,000,000–1,990,000	362(33.5)	50.73 ± 14.35	264(72.9)
	2,000,000–2,990,000	178(16.5)	48.11 ± 11.25	119(66.9)
	≥3,000,000	198(18.4)	43.26 ± 8.15	81(40.9)
	Neglect to answer	29(2.7)	47.97 ± 11.13	16(55.2)
**Occupation**				
	Managers, professionals and experts	174(16.1)	43.17 ± 9.27	108(45.6)
	Engineers, semi-experts	63(5.8)	41.11 ± 10.86	27(42.9)
	Office workers	100(9.3)	45.67 ± 11.16	52(52.0)
	Service and sales workers	157(14.6)	53.36 ± 17.26	111(70.7)
	Skilled agricultural, forestry and fishery workers	18(1.7)	45.39 ± 16.15	194(69.0)
	Craft and related trades workers	131(12.1)	47.43 ± 11.94	92(70.2)
	Machine operator, assembly workers	132(12.2)	49.80 ± 13.93	91(63.5)
	Elementary workers	304(28.2)	48.68 ± 15.80	193(63.5)
**Form of employment**				
	Regular	772(71.5)	48.56 ± 12.78	491(63.6)
	Temporary or part-time	307(28.5)	45.46 ± 17.13	167(54.4)
**BMI (kg/m**^**2**^**)**				
	<25	870(80.6)	47.63 ± 13.76	538(61.8)
	≥25	209(19.4)	47.89 ± 15.99	120(57.4)
**Alcohol habit**				
	No	453(42.0)	48.35 ± 14.61	293(64.7)
	Yes	687(57.2)	47.20 ± 13.91	365(58.3)
**Smoking habit**				
	Past or nonsmoker	735(68.1)	47.67 ± 14.73	455(61.9)
	Current smoker	344(31.9)	47.69 ± 15.20	203(59.0)
**Regular Exercise**				
	Once a week or more	433(40.1)	45.14 ± 13.34	224(51.7)
	Less than once a week	646(59.9)	49.38 ± 14.53	434(67.2)
**Working days per week**				
	≤5 days	539(50.0)	40.92 ± 13.15	158(29.3)
	≥6 days	540(50.0)	54.43 ± 11.84	500(92.6)
**Self-reported hypertension during follow-up**				
	None	994(92.1)	47.45 ± 14.00	597(60.1)
	Hypertension	85(7.9)	50.38 ± 16.30	61(71.8)
**Total**		1079(100.0)	47.68 ± 14.21	658(61.0)

Table 2 shows the general characteristics of all subjects categorized by working hours per week. In the ≥61 h group, the proportion of female subjects tended to be higher than that in the other groups. The higher the number of working hours per week, the lower the level of education. In the ≤40 h group, managers, professionals, and expert workers comprised higher proportions than in the other groups classified by working hours. In the ≥61 h group, the proportions of elementary workers and service and sales workers were also higher. The proportion of subjects who worked ≥6 days a week in the 51- to 60-h group was 89.3%, which was a higher proportion than in the other groups.

**Table 2:** General characteristics of the selected population according to number of working hours per week

**Characteristics**		**Number of working hours per week**				
		**≤40**	**41–50**	**51–60**	**≥61**	**p**
		**(n = 476)**	**(n = 366)**	**(n = 199)**	**(n = 165)**	
**Male, n (%)**		286 (67.9)	223 (66.2)	117 (65.7)	77 (53.8)	0.022
**Age, years (SD)**		52.1 (5.8)	52.0 (5.7)	52.3 (6.6)	54.1 (7.1)	*0.003
**Education**						
	Elementary school graduation or less	83 (19.7)	51 (15.1)	39 (21.9)	45 (31.5)	<0.001
	Middle school graduation	65 (15.4)	70 (20.8)	39 (21.9)	43 (30.1)	
	High school graduation	156 (37.1)	148 (43.9)	80 (44.9)	47 (32.9)	
	College graduation or beyond	117 (27.8)	68 (20.2)	80 (11.2)	8 (5.6)	
**Income (won)**						
	≤990,000	134 (31.8)	76 (22.6)	41 (23.0)	61 (42.7)	<0.001
	1,000,000-1,990,000	98 (23.3)	117 (34.7)	86 (48.3)	61 (42.7)	
	2,000,000-2,990,000	59 (14.0)	71 (21.1)	33 (18.5)	15 (10.5)	
	≥3,000,000	117 (27.8)	64 (19.0)	13 (7.3)	4 (2.8)	
	Neglect to answer	13 (3.1)	9 (2.7)	5 (2.8)	2 (1.4)	
**Occupation**						
	Managers, professional and experts	93 (22.1)	62 (18.4)	16 (9.0)	3 (2.1)	<0.001
	Engineers, semi-experts	36 (8.6)	22 (6.5)	3 (1.7)	2 (1.4)	
	Office workers	48 (11.4)	32 (9.5)	16 (9.0)	4 (2.8)	
	Service and sales workers	46 (10.9)	32 (9.5)	30 (16.9)	49 (34.3)	
	Skilled agricultural, forestry and fisheries workers	7 (1.7)	5 (1.6)	3 (1.7)	3 (1.7)	
	Craft and related trades workers	39 (9.3)	50 (14.8)	33 (18.5)	9 (6.3)	
	Machine operators, assembly workers	41 (9.7)	43 (12.8)	26 (14.6)	22 (15.4)	
	Elementary workers	111 (26.4)	91 (27.0)	51 (28.7)	51 (35.7)	
**Form of employment**						
	Regular	281 (66.7)	264 (78.3)	138 (77.5)	89 (62.2)	<0.001
	Temporary or part-time	140 (33.3)	73 (21.7)	40 (22.5)	54 (37.8)	
**BMI**						
	kg/m^2^ (SD)	23.25 (2.8)	23.2 (2.5)	23.0 (2.5)	23.0 (2.8)	*0.731
**Alcohol habit**						
	Yes, n (%)	261 (62.0)	188 (55.8)	99 (55.6)	78 (54.5)	0.208
**Smoking habit**						
	Current smoker, n (%)	141 (33.5)	95 (28.2)	55 (30.9)	53 (37.1)	0.215
**Exercise**						
	Less than once a week, n(%)	212 (50.4)	200 (59.3)	130 (73.0)	104 (72.7)	<0.001
**Working days per week**						
	≥6 days, n (%)	40 (9.5)	230 (68.2)	159 (89.3)	111 (77.6)	<0.001
**Self-reported hypertension during follow-up**						
	Yes, n (%)	24 (5.7)	31 (9.2)	14 (7.9)	16 (11.2)	0.126

*p-value of Kruskal–Wallis test. Others were calculated by Pearson’s chi-squared test.

Table 3 shows the results of the Cox proportional hazards models fitted to evaluate the association between work hours and the incidence of self-reported hypertension. The mean follow-up time was 3.68 years (median, 4.00 years). Among all subjects, 85 initially free of hypertension reported a medical diagnosis of hypertension (males, 53; females, 32). The cumulative incidence in these study subjects was 7.9% during the follow-up period.

**Table 3:** Number of working hours per week and the risk of self-reported hypertension in 1079 normotensive workers

	**Number of working hours per week**				
	**≤40**	**41–50**	**51–60**	**≥61**	**p for trend**
	**(n = 421)**	**(n = 337)**	**(n = 178)**	**(n = 143)**	
No. of onsets	24	31	14	16	
Follow-up periods (person-months)	1569.12	1230.38	646.68	528.03	
Rate per 1000 person-months	15.30	25.20	21.65	30.30	
Model 1 (95% CI)	1.00 (reference)	1.64 (0.97–2.75)	1.50 (0.79–2.84)	1.99 (1.06–3.73)	0.036
Model 2 (95% CI)	1.00 (reference)	1.63 (0.97–2.74)	1.46 (0.77–2.78)	1.81 (0.95–3.44)	0.074
Model 3 (95% CI)	1.00 (reference)	1.79 (1.06–3.03)	1.72 (0.89–3.30)	1.95 (1.02–3.73)	0.033
Model 4 (95% CI)	1.00 (reference)	2.20 (1.19–4.06)	2.40 (1.07–5.39)	2.87 (1.33–6.20)	0.013

Model 1 was not adjusted.

Model 2 was adjusted for age and sex.

Model 3 was adjusted for age, sex and health-related factors including smoking habit, alcohol habit, regular exercise, and body mass index (BMI).

Model 4 was adjusted for age, sex, health-related factors, education and occupational factors including income, occupation, form of emplyment, and working days per week.

All hazard ratios were calculated using a Cox proportional hazards model with weighted cases.

CI = Confidence interval.

Among the groups classified by work hours, the highest number of subjects worked ≤40 h per week (421 subjects), and the lowest number worked ≥61 h (143 subjects). The cumulative incidence of hypertension was highest in the 41- to 50-h group (31 subjects) and lowest in the 51- to 60-h group (14 subjects).

Using the Cox proportional hazard models as a reference for the ≤40-h group, in the case of Model 1, which was not adjusted, the ≥61-h group was statistically significant (hazard ratio [HR], 1.99; 95% confidence interval [CI], 1.06–3.73). There was no statistically significant group in Model 2, which adjusted for age and sex. In Model 3, which adjusted for age, sex, and health-related factors, the 41 to 50-h group (HR, 1.79; CI, 1.06–3.03) and ≥61-h group (HR, 1.95; CI, 1.02–3.73) were statistically significant. Model 4 adjusted for age, sex, health-related factors, education, and occupational factors including income, occupation, form of employment, and number of days worked per week. The hazard ratios were 41–50 h (HR, 2.20; CI, 1.19–4.06), 51–60 h (HR, 2.40; CI, 1.07–5.39), and ≥61 h (HR, 2.87; CI, 1.33–6.20); thus, there was whole-group statistical significance.

A Cox proportional hazards model was additionally developed to observe the association between working hours and self-reported hypertension after gender stratification (Table 4). Model 1_s_ was not adjusted. Model 2_s_ was adjusted for age. Model 3_s_ was adjusted for age, and health-related factors. Model 4_s_ was adjusted for age, health-related factors, education, and occupational factors. Except for the ≥61-h group of Model 4_s_, the hazard ratio of the females tended to be higher than that of the males.

**Table 4:** Number of working hours per week and the risk of self-reported hypertension stratified by gender

**Sex**	**Models**	**Number of working hours per week**				**p for trend**
		**≤40**	**41–50**	**51–60**	**≥61**	
		**(n = 421)**	**(n = 337)**	**(n = 178)**	**(n = 143)**	
Males	Number of workers	286	223	117	77	
	Model 1_s_ (95% CI)	1.00 (reference)	1.61 (0.85-3.03)	1.49 (0.68-3.26)	1.79 (0.77-4.15)	0.151
	Model 2_s_ (95% CI)	1.00 (reference)	1.61 (0.85-3.03)	1.50 (0.69-3.29)	1.61 (0.68-3.83)	0.217
	Model 3_s_ (95% CI)	1.00 (reference)	1.73 (0.91-3.28)	1.72 (0.78-3.79)	1.63 (0.68-3.93)	0.163
	Model 4_s_ (95% CI)	1.00 (reference)	2.19 (1.03-4.63)	2.56 (0.91-7.15)	2.54 (0.90-7.15)	0.093
Females	Number of workers	135	114	61	66	
	Model 1_s_ (95% CI)	1.00 (reference)	2.23 (0.83-6.03)	2.27 (0.73-7.05)	2.35 (0.79-6.99)	0.128
	Model 2_s_ (95% CI)	1.00 (reference)	2.01 (0.74-5.47)	2.21 (0.71-6.90)	2.15 (0.72-6.42)	0.166
	Model 3_s_ (95% CI)	1.00 (reference)	2.29 (0.83-6.29)	2.76 (0.86-8.85)	2.76 (0.90-8.43)	0.112
	Model 4_s_ (95% CI)	1.00 (reference)	2.86 (0.79-10.34)	3.13 (0.74-13.17)	2.19 (0.49-9.84)	0.066

Model 1_s_ was not adjusted.

Model 2_s_ was adjusted for age.

Model 3_s_ was adjusted for age, and health-related factors including smoking habit, alcohol habit, regular exercise, and body mass index(BMI).

Model 4_s_ was adjusted for age, health-related factors, education and occupational factors including income, occupation, form of employment, and working days per week.

All hazard ratios were calculated using a Cox proportional hazards model with weight cases.

CI = Confidence interval.

## Discussion

In this study, we attempted to identify the relationship between long working hours and self-reported hypertension. In Model 4, which adjusted for all selected variables, we identified a positive association between the 41 to 50-h, 51 to 60-h, and ≥61-h groups and the incidence of self-reported hypertension. Also in Model 4, the p for trend, which showed statistical significance (p = 0.013), suggested that the risk of self-reported hypertension increases as the number of working hours increases (Table 3). Therefore, we concluded that long working hours is an independent risk factor for hypertension among middle-aged and older wage workers.

It is difficult to define ‘long working hours’, because the government definition varies from nation to nation. Bannai et al. [27] noted that the standard numbers of working hours of Korea, the US, France, and Denmark are 40 [28], 40 [29], 37 [29], and 35 hours/week, respectively. This resulted in the differences among the definitions for ‘long working hours’ of the researchers of each country. Our study defined ‘long working hours’ as over 40 h/week based upon the Korean Labor Standards Act because the subjects were Korean workers.

Our study found a higher hazard ratio in female workers than male ones except in one group; however, there were no statistically significant groups (Table 4). In female workers, the proportion of those in the service and sales group, for which the average number of working hours is the highest (Table 1), was much greater than the proportion of service and sales workers among males: 37.0% versus 2.6%, respectively. Because of the heterogeneity of distribution, the proportion of female workers in the ≥61 h group was the highest among the groups classified by number of working hours (Table 2). This shows that the working conditions of female workers in Korea are worse than those of males. Meanwhile, gender-based physiological changes due to long working hours might not be the same in males and females [30]. Female workers usually have to bear the burden of job and domestic work. Actually working burden of female workers is higher than male’s one [31].

Our results are consistent with those of earlier studies. Nakamura et al. [32] showed that extensive overtime work was associated with increased blood pressure in normotensive male assembly-line workers. However, there was a limitation in terms of the lack of investigation of an association for clerks, engineers, and engineers/special technicians. Artazcoz et al. [33] showed that long work hours are associated with hypertension (adjusted odds ratio, 2.25; 95% CI, 1.17–4.32) among female workers in a cross-sectional study. Further research on sex differences in the workplace in Korea is necessary. Yang et al. [20] showed that individuals who worked 40, 41 to 50, and >50 h per week were 1.14-fold (95% CI, 1.01–1.28), 1.17-fold (95% CI, 1.04–1.33), and 1.29-fold (95% CI, 1.10–1.52) more likely to have self-reported hypertension, respectively, than were individuals who worked 11 to 39 h per week in a cross-sectional study. Even though that study had a cross-sectional study design, but its findings are still meaningful because it targeted subjects in all age groups above the age of 18 years as well as all occupational groups. Hayashi et al. [9] found that within a group of white-collar workers followed longitudinally, blood pressure was significantly higher and number of sleep hours was significantly lower when working overtime (average overtime of 96 h/month) than those in a “control” period (average overtime of 43 h/month). Ambulatory monitoring methods were used in this study, which can be viewed as more valid due to the higher number of readings and lack of observer bias compared with any other method. Iwasaki et al. [10] showed significantly higher systolic blood pressure among salesmen aged 50 to 60 years who spent >61 h per week commuting and working than among those who spent <57 h. This study is worth comparing to ours in that the age group with significance is similar. However, there are differences in that the target subject occupation was limited to salesmen and the cutoff time with significance was 61 h.

On the other hand, several Japanese studies have reported negative associations. Nakanishi et al. [21] showed that long working hours are negatively associated with the risk of hypertension among male workers. This 5-year prospective cohort study was designed to directly measure blood pressure through serial annual health examinations. Nevertheless, it showed dissimilarities from our study and has limited generalizability because the subjects were only white-collar workers in Japan and were aged 35 to 54 years. Blue-collar workers reportedly have a higher risk of hypertension than do white-collar workers [34]. Therefore, it is possible that the influence of overtime work on the risk of hypertension might be underestimated. In addition, the authors defined hypertension as a systolic blood pressure of ≥160 mmHg and diastolic blood pressure of ≥95 mmHg, but these values have not been used since 1999 when the World Health Organization adopted a systolic blood pressure of ≥140 mmHg or diastolic blood pressure of ≥90 mmHg as cutoff value for hypertension [25]. Another study by Nakanishi et al. [3] found that working ≥10 h per day was an independent negative factor associated with the development of hypertension in a 3-year follow-up study of middle-aged Japanese male office workers. Similarly, their study was limited in that the subjects were restricted to male office workers and they were not able to control for all occupational groups. Wada et al. [22] showed that workers whose mean overtime was ≥50 h per month had lower risks of developing definite hypertension and high BMI. The advantages of this study were that blood pressure was directly examined and definite hypertension was measured; however, there were differences from our study in that they examined only male subjects and did not adjust for confounding factors other than age.

Meanwhile, some studies have shown no association. For instance, data collected in the Minnesota Heart Survey [24] revealed no association between job experience, including working hours, and blood pressure. Pimenta et al. [25] also showed no association between work hours or “total activity hours” and the incidence of hypertension among a cohort of Spanish university graduates. The limitation of this study was that the population that was studied comprised university graduates, who are more likely to follow healthier lifestyles that could decrease their risk of developing hypertension. In other words, selection bias was not controlled for.

Our study addressed several of the above mentioned limitations of earlier studies. First, various occupations including blue-collar and white-collar were controlled for so that our study subjects were representative of Korean wage workers. Second, we considered the temporality of the association by using prospective data, in contrast to other cross-sectional studies. Third, we adjusted for occupation- and health-related factors, for which other studies did not control.

However, we unexpectedly observed a decrease in the hazard ratios among subjects who worked 51–60 h per week in Models 1 through 3. Heterogeneity of occupational factors seemed to be an influential factor in those three models because the decrease disappeared after adjusting for all factors, including occupational factors. The exact reason for this phenomenon is elusive. However, we propose possible explanations based on the general characteristics of the subjects. First, the working days per week might have played an important role. Even with the same working hours, working 5 days or 6 days per week could affect the worker differently. Second, adjustment for type of occupation could have changed the hazard ratio of Model 4 because the average number of working hours varies according to the occupation.

On the basis of several theories of the mechanism of hypertension secondary to long working hours, a plausible explanation of our research results can be proposed. First, overtime work may act directly as a stressor. Belkic et al. [35] showed that normotensive workers developed increased blood pressure in the workplace. Second, overtime work may increase exposure to other workplace hazards such as job stress and effort–reward imbalance. Permanent physiological changes can develop, and hypertension can develop secondary to these factors [36]. Third, overtime work may promote unhealthy behaviors such as drinking or smoking. However, actual research results are mixed in terms of both positive and negative associations [37]-[39]. Fourth, overtime work may be associated with fatigue and shorter sleep hours. Sleep deprivation exacerbates the activity of the sympathetic nervous system, increases the heart rate, and elevates the blood pressure. This hypothesis is supported by the fact that <6 h of sleep a day was associated with a higher risk of heart disease compared with a normal sleep group in one study [40]. In addition, lack of sleep is involved in a shorter time available for recovery and is correlated with disruption of physiological processes [8].

The limitations of this study were as follows. First, the incidence of hypertension was measured by self-reported hypertension questionnaires and not accurately diagnosed by directly checking blood pressure. However, self-reported hypertension can be treated as a comparable measurement of “awareness of hypertension” [41]. Other studies have shown that the proportion of self-reported hypertension was usually lower than the measured blood pressure [42],[43]. Therefore, self-reported hypertension seems to be an unbiased underestimation of true hypertension [20]. Selem et al. [44] showed that self-reported hypertension is valid in adults and elderly individuals and is thus an appropriate indicator for surveillance of hypertension prevalence in the absence of blood pressure measurement. Second, with the exception of BMI, smoking history, and drinking habit, we could not adjust for other risk factors of hypertension (e.g., family history, high-salt diet, high-potassium diet, etc.) because of data limitations. Third, all subjects’ were ≥45 years of age. This study could not target workers of all age groups, so well-designed studies using larger sample sizes and a wider variety of age would be valuable in the future. Fourth, we did not consider the variation of working hours from 2006 to 2010; the number of working hours may have changed with workers’ changing positions or departments.

## Conclusion

Previous studies were inconclusive on the association of long working hours and hypertension. However, our findings suggest that the risk of self-reported hypertension increases as the number of working hours increases. Thus, long working hours (≥41 h) may be an independent factor associated with hypertension among middle-aged and older wage workers. Working hour management might thus be essential for prevention of hypertension among workers.

## Competing interests

The authors declare that they have no competing interests.

## Authors’ contributions

MYK and DHY designed the research. BM collected the data. MYK and DHY performed the statistical analysis. MYK, DHY, SC, and DMP interpreted the data. DHY and SC wrote the manuscript. All of the authors read and approved the final manuscript.
